# Meeting of the Representatives of American Medical Colleges

**Published:** 1876-06

**Authors:** 


### Meeting of Representatives of American Medical Colleges.

We publish elsewhere, page 424, a report of the meeting of delegates from American Medical Colleges, held in Philadelphia, June 2d and 3d. We have not been disappointed in the results of this Convention, as we did not expect much to be accomplished. An organization of this kind, where the delegates are not empowered to commit the faculties which they represent, to the decisions of the Association, and whose first action after organization is to pass a resolution distinctly stating that the action of the convention shall not be considered binding unless endorsed by the faculties represented, can not be expected to accomplish much. Aside from expressing the opinion of those present in regard to various matters of educational reform, we do not see how the convention expected to do anything.

The first question discussed and resolved upon is in reference to the benificiary system and its abuses. We think that aside from one or two schools who have adopted a system of beneficiary scholarships and *advertised for applicants*, there is no one the profession who is in favor of placing a low estimate upon the value of a medical education. A young man, if thoroughly in earnest, will find means to obtain his education, and prize it the higher when he has won it at the cost of some sacrifice.

Question number two every one will answer in the negative, who does not want to see the medical profession make a retrograde instead of an advance move. Under the present or under any method of medical teaching, three years is a period sufficiently short for the most ardent seeker after a diploma, and those who propose to shorten the period of study should not receive the recognition of the profession. Of these two questions as well as of questions three, five, six and seven, we can simply say that in the minds of every honest, well-meaning physician they were decided as the convention decided them, long ago, and that an association of delegates of American Medical Colleges should consider it necessary to seriously consider and vote upon them is an imputation upon the honesty of the teachers of medicine in the United States. As the subject of uniform college fees is held over until 1877, we will reserve what we have to say on that subject until later. Near the close of the convention a resolution was introduced saying that no degree should be conferred without examination in person of the candidate. From the tone of this resolution one not familiar with the subject would infer that medical colleges were in the habit of conferring degrees without examination of the candidate. We cannot believe that Jefferson College with which the mover of this resolution is connected, can be guilty of such a thing, and for the honor of the profession and good of humanity, we earnestly hope that that no medical school, under any circumstances, could be induced to confer degrees without examination. The convention may, upon the whole, be called a failure as far as results are concerned. We hope that when it next meets that its work will be of a more practical kind, such, for instance, as the best method of introducing into all medical schools a *thorough, graded,* course of instruction.

